# DNA supercoiling, a critical signal regulating the basal expression of the *lac* operon in *Escherichia coli*

**DOI:** 10.1038/srep19243

**Published:** 2016-01-14

**Authors:** Geraldine Fulcrand, Samantha Dages, Xiaoduo Zhi, Prem Chapagain, Bernard S. Gerstman, David Dunlap, Fenfei Leng

**Affiliations:** 1 Biomolecular Sciences Institute, Florida International University, Miami, FL 33199; 2 Department of Chemistry & Biochemistry, Florida International University, Miami, FL 33199; 3 Department of Physics, Florida International University, Miami, FL 33199; 4 Department of Physics, Emory University, Atlanta, GA 30322.

## Abstract

*Escherichia coli lac* repressor (LacI) is a paradigmatic transcriptional factor that controls the expression of *lacZYA* in the *lac* operon. This tetrameric protein specifically binds to the *O1*, *O2* and *O3* operators of the *lac* operon and forms a DNA loop to repress transcription from the adjacent *lac* promoter. In this article, we demonstrate that upon binding to the *O1* and *O2* operators at their native positions LacI constrains three (−) supercoils within the 401-bp DNA loop of the *lac* promoter and forms a topological barrier. The stability of LacI-mediated DNA topological barriers is directly proportional to its DNA binding affinity. However, we find that DNA supercoiling modulates the basal expression from the *lac* operon in *E. coli*. Our results are consistent with the hypothesis that LacI functions as a topological barrier to constrain free, unconstrained (−) supercoils within the 401-bp DNA loop of the *lac* promoter. These constrained (−) supercoils enhance LacI’s DNA-binding affinity and thereby the repression of the promoter. Thus, LacI binding is superhelically modulated to control the expression of *lacZYA* in the *lac operon* under varying growth conditions.

The *Escherichia coli lac* operon is a paradigm for transcriptional regulation in prokaryotes^1^,^2^. The primary regulator is the *lac* repressor (LacI) that specifically binds to operators at the *lac* promoter region^3^ and inhibits transcription from the *lac* promoter in the absence of an inducer^4^. There are three operators: the primary *O1* operator located 11 bp downstream of the transcription-starting site from the *lac* promoter^1^,^4^ and two auxiliary *O2* and *O3* operators located at 412 and −82 bp, respectively, of the promoter region^4^. Since only about ten copies of LacI tetramer are simultaneously present in each *E. coli* cell^5^, the function of *O2* and *O3* is to increase the local concentration of LacI around the *lac* promoter^6^ and therefore increase the efficiency of repression^4^,^7^. The second regulator is the cAMP-CRP complex (CRP refers to cAMP receptor protein) that binds to the CRP binding site centered at position −61.5 bp upstream from the transcription-starting site of the *lac* promoter^8^. Binding of cAMP-CRP complex to its binding site at the *lac* promoter strongly activates transcription initiation from the *E. coli lac* promoter^9^. The deletion of *crp* from the chromosome or the CRP-binding site from the *lac* promoter greatly reduced the promoter strength in the presence of an inducer^10^,^11^. This dual control of the *lac* promoter by LacI and CRP ensures the maximal repression in the absence of an inducer^12^. However, when glucose level is low and lactose level is high, the operon is fully activated^13^,^14^.

In *Escherichia coli*, DNA is typically (−) supercoiled^15^,^16^,^17^. In fact, DNA supercoiling plays critical roles in several essential DNA metabolic pathways, such as DNA replication, transcription, and recombination^15^,^16^,^17^. DNA supercoiling is also involved in the regulation of the *lac* operon. For instance, it has been demonstrated that negative supercoiling enhances the binding of LacI to *lac* operators^18^,^19^ and promotes LacI-mediated DNA looping^20^,^21^,^22^. Additionally, LacI induces supercoiling within the LacI-*lac O1* complexes and retains certain superhelical energy in the complexes as well^18^,^23^,^24^. Recently, we showed that binding of LacI tetramers to tandem copies of *lac O1* operators in different locations of a DNA molecule separated a supercoiled DNA molecule into two topologically independent, looped domains^25^. These supercoiled domains are highly stable and supercoils diffuse through the LacI loop closure with a half-life of 112 min^25^. A topological barrier model in which nucleoprotein complexes confine DNA supercoils to localized regions is consistent with these results^25^. These results also led us to pose the following questions: Is LacI able to form topological barriers and constrain supercoils in the *lac* promoter region upon binding to the *O1*, *O2*, and *O3* operators at their native positions? If so, is such trapped superhelicity biologically significant? Here utilizing combined approaches of biochemical assays, bacterial genetics, and atomic force microscopy, we show that upon simultaneously binding the *O1* and *O2* operators LacI forms a topological barrier that divides a supercoiled plasmid DNA molecule containing the *lac* promoter region into distinct topological domains. We also demonstrate that DNA supercoiling is an important modulator of the basal level of gene expression from the *lac* operon and that the LacI-mediated topological barrier plays an essential role in this process.

## Results

### The stability of LacI-mediated DNA topological barriers is correlated with their DNA binding affinity

**Figure 1:**
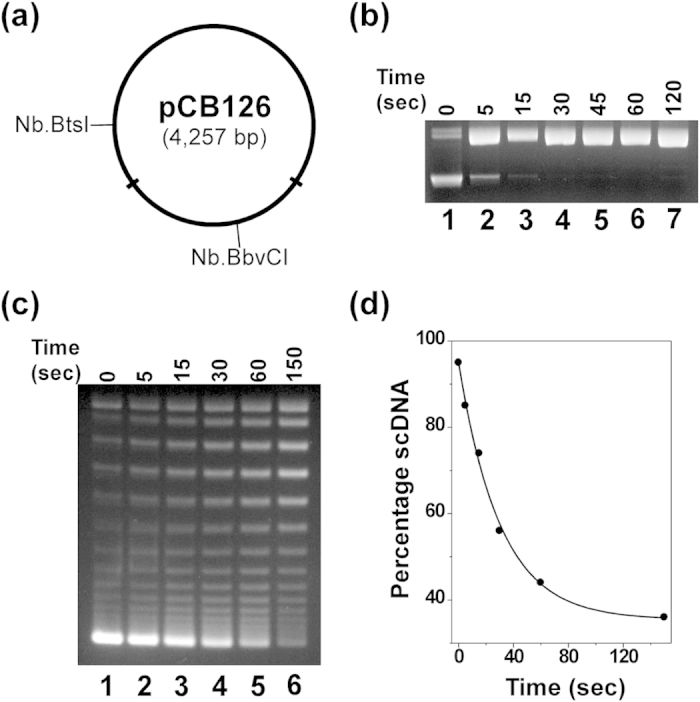
One molecule of LacI tetramer divided a supercoiled DNA molecule plasmid pCB126 into two independent topological domains. (**a**) Plasmid pCB126 carrying two *lac O1* operators in two different locations was constructed as detailed in Methods. (**b**) The nicking enzyme Nt.BbvCI was able to rapidly digest pCB126. Time course of enzyme digestion of pCB126 using 16 units of Nt.BbvCI in 1 × NEBuffer 4 at 37 °C. Lane 1 contained the undigested scDNA. (**c**) Time course of DNA supercoiling diffusion in the presence of LacI. The DNA-nicking assays were performed as described under Methods. Each reaction mixture (320 μL) contained 0.156 nM of pCB126, 2.5 nM of LacI, and 16 units of Nt.BbvCI. The reactions were incubated at 37 °C for the time indicated. Then a large excess of a double-stranded oligonucleotide contain an Nt.BbvCI recognition site was added to the reaction mixture to inhibit the restriction enzyme activities. The nicked DNA templates were ligated by T4 DNA ligase in the presence of 1 mM of ATP at 37 °C for 5 min and the reactions were terminated by phenol extraction. The DNA molecules were isolated and subjected to agarose gel electrophoresis. (**d**) Quantification analysis of the time course. The percentage of supercoiled DNA was plotted against the reaction time. The curve was generated by fitting the data to a 1st-order rate equation to yield a rate constant of 0.016 sec^−1^ and a t_1/2_ of 52 sec.

Our previous results showed that two LacI tetramers bound to the four-*lac O1* operators of plasmid pCB115 and formed two highly stable LacI-*lac O1* nucleoprotein complexes^25^. These established a topological barrier and divided the supercoiled DNA molecule into two topological domains, a nicked & relaxed domain and a supercoiled domain^25^. Using similar approaches (Supplementary Fig. S1), we determined the stability of LacI-mediated topological barriers for plasmids pCB116, pCB108, and pCB109 that contain, respectively, 8, 16, and 32 *lac O1* operators in two different locations spaced ~1.2 and 2.9 kb apart (Supplementary Fig. S2). In the absence of IPTG, the multiple LacI-mediated topological barriers have similar t_1/2_ of ~120 min (Table 1 and Supplementary Fig. S3a–c). The only exception is the DNA topological barrier for plasmid pCB126 with a t_1/2_ of 0.87 ± 0.33 min that results from binding of one molecule of LacI to two lac O1 operators (Table 1). Intriguingly, even in the presence of IPTG, LacI was able to form multiple LacI-*lac O1* complexes and block supercoil diffusion (Table 1 and Supplementary Fig. S3d–g). These results suggest that IPTG is not capable of dissociating LacI from the tandem copies of *lac O1* operators, which is consistent with previously published results^26^. In order to detect and determine the stability of LacI-mediated topological barriers for plasmid pCB126 that has single copies of *lac O1* operators (Fig. 1a), significantly more Nt.BbvCI was used, which was able to nick more than 90% of supercoiled pCB126 molecules within 5 seconds (Fig. 1b and Supplementary Fig. S4a,b). In this way, we determined the t_1/2_ of the topological barrier mediated by one LacI tetramer to be 52 ± 12 sec (0.87 ± 0.33 min) (Fig. 1c,d and Table 1). Although the t_1/2_ of the LacI-mediated topological barrier on pCB126 is significantly lower than that of the multiple LacI-mediated topological barriers, one LacI tetramer significantly blocked supercoil diffusion, which otherwise would be on the order of milliseconds^27^,^28^.

Next, we constructed a series of plasmid DNA templates that contain different pairs of *O1*, *O2*, *O3*, and *Os* operators (Supplementary Fig. S2; *Os* represents the symmetric *lac* operator^29^). We then determined t_1/2_ values of the LacI-mediated topological barriers for these DNA templates. Our results in Table 2 clearly demonstrat that the stability of the LacI-mediated topological barriers is correlated with the affinity of LacI binding for different *lac* operators (Supplementary Fig. S4c,d and Supplementary Fig. S5; Supplementary Table S1). For instance, the t_1/2_ of the LacI-mediated topological barriers upon binding to two *Os* operators on pOsOs was determined to be 29.1 ± 4.6 min (Supplementary Fig. S5 and Supplementary Table S1). In contrast, the t_1/2_ of the LacI-mediated topological barrier for two *O3* operators on pO3O3 was determined to be only approximately 5.5 ± 1.2 sec. We also determined the t_1/2_ values of DNA topological barriers generated from binding of LacI mutants^30^ LacI-Gly^58+1^, LacI-Gly^60+1^, LacI-Gly^60+2^, and LacI-Gly^60+3^ to the two *Os* sites on pOsOs. Again, our results showed that the t_1/2_ values of the LacI-mediated topological barriers scale proportionally with the DNA binding affinities of these LacI mutants (Supplementary Table S2).

### LacI forms a DNA topological barrier upon binding to lac operators in the *lac* operon

**Figure 2:**
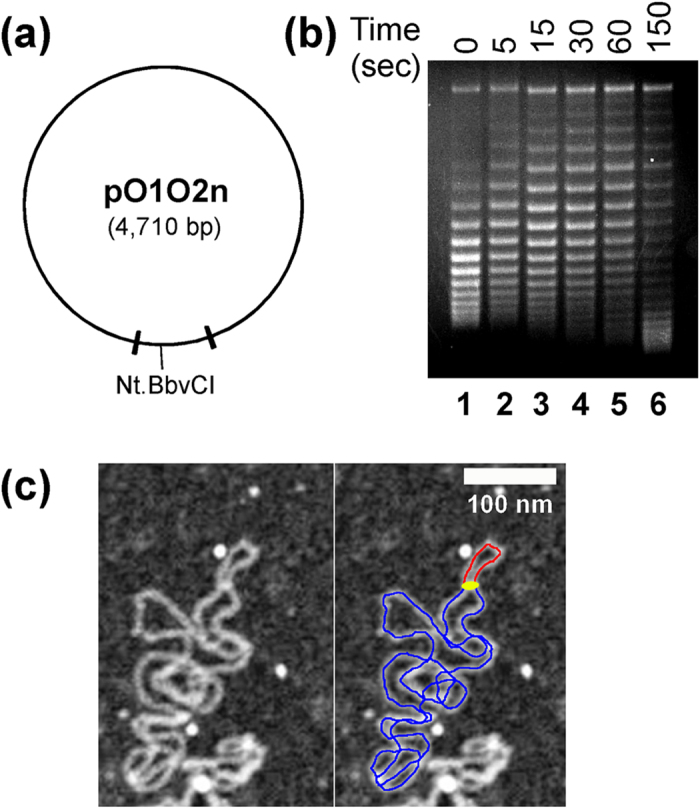
LacI was able to form a DNA topological barrier upon binding to the *O1* and *O2* operators at their native positions and constrain 3 supercoils to the 401 bp DNA loop. (**a**) Plasmid pO1O2n carrying the *lac O1* and *O2* operators at their native positions of the *lac* promoter was constructed as detailed in Methods. (**b**) The DNA-nicking assays (time course) using supercoiled plasmid pO1O2n (σ = ~−0.06) were performed as described under Methods. The reaction mixtures in the presence of LacI were incubated at 37 °C for the time indicated. The DNA topoisomers were isolated and subjected to agarose gel electrophoresis in the presence of 1.5 μg/ml of chloroquine. LacI was able to constrain 2.8 ± 0.7 supercoils within the 401 bp LacI-mediated DNA loop. (**c**) AFM images demonstrate that LacI divided a supercoiled DNA molecule (plasmids pOsOs401) into two independent topological domains: a 401 bp relaxed domain and a large supercoiled domain. The AFM imaging experiments were performed as described in ref. 25. The right panel is a traced image alongside the original image (left panel) indicating the supercoiled domain (blue trace), the 401 bp relaxed domain (red trace), and the LacI molecule (yellow oval).

We constructed three unique plasmids harboring the natural *lac* promoter including either active or mutated *O1*, *O2*, and *O3* at their native positions (Fig. 2 and Supplementary Fig. S6). We also inserted a nicking endonuclease Nt.BbvCI recognition site between the *O1* and *O2* operators. Moreover, we made two plasmid DNA templates, pOsOs401 and pOsOs493, with two *Os* operators replacing either the *O1* & *O2* operators of plasmid pO1O2n or the *O2* & *O3* operators of plasmid pO3O2n (Supplementary Fig. S6). After construction of these plasmids, we employed the DNA-nicking assay to examine the stability of LacI-mediated topological barriers and to determine how many supercoils were trapped in the 401 and 493 bp DNA loops upon the formation of topological barriers (the initial supercoiling density of these plasmid DNA templates was approximately −0.06). Our results are shown in Fig. 2b, Supplementary Fig. S7, and Table 2. As expected, LacI was able to form topological barriers upon binding to *O1*, *O2*, and *O3* at their native positions and divided the supercoiled DNA molecules into two independent topological domains, a supercoiled domain and a nicked, relaxed domain (Fig. 2c). LacI was also able to trap ~3 to 4 (−) supercoils within the 401 and 493 bp DNA loops, respectively (Fig. 2b and Supplementary Fig. S7; Table 2). Intriguingly, the stability of the LacI-mediated topological barriers quantified by the exponential decay constants for the diffusion of supercoiling (t_1/2_) were inversely related to the size of the nicked domain. For instance, the t_1/2_ of the LacI-mediated topological barrier for pO1O2n with a 401 bp nicked domain is significantly greater than that for pO1O2 with a 1.2 kb nicked domain (Table 2 and Supplementary Table S1). These results are consistent with our recent results for λ repressor mediated topological barriers^31^.

We also used atomic force microscopy (AFM) to examine how one molecule of LacI divided a supercoiled DNA molecule into two independent topological domains. We used supercoiled plasmid pOsOs401 and the DNA nicking method for our AFM studies. After supercoiled pOsOs401 was nicked by Nt.BbvCI in the presence of LacI, the LacI-plasmid complexes were deposited on a freshly cleaved mica surface and imaged. Our results, summarized in Fig. 2c and Table 3, clearly demonstrated that one molecule of LacI binding to the *lac* operators at the native positions divided a supercoiled DNA molecule into two independent topological domains. In the absence of LacI, the average contour length of the DNA molecules was measured to be 1,584.4 ± 78.2 nm (Table 3). For B-form DNA with 0.34 nm per bp, this length was calculated to be 4,660 ± 230 bp, which matches the plasmid sequence length of 4,595 bp. The LacI molecule divided the DNA molecule into a 1,441.2 ± 50.3 nm (4,239 ± 148 bp) supercoiled domain and a 140.9 ± 23.8 nm (414 ± 70 bp) relaxed domain. These lengths are consistent with estimates based on the DNA sequences of the two topological domains (Table 3).

### DNA supercoiling is an important signal to control the basal expression of the *lac* operon in *E. coli*

**Figure 3:**
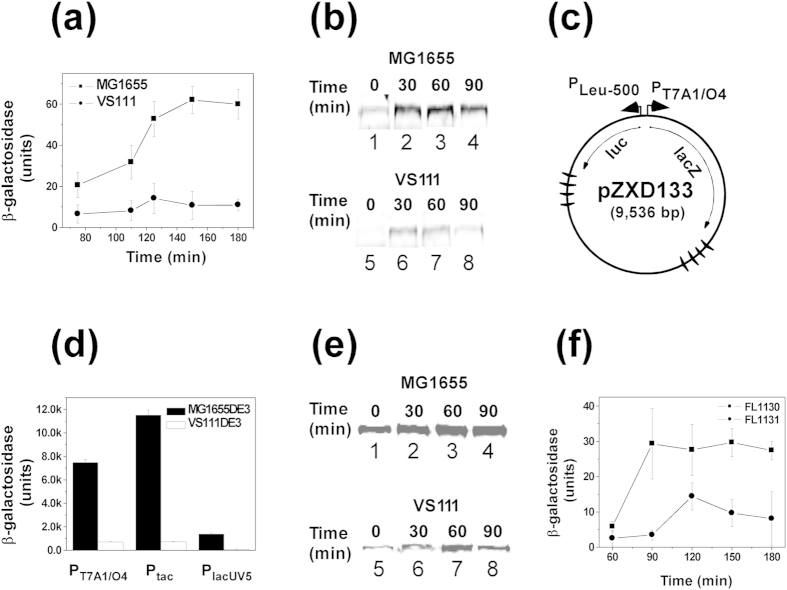
The basal expression of β-galactosidase in the absence of an inducer for *E. coli* wildtype strain *MG1655* and the isogenic *topA* strain *VS111*. (**a**) The β-galactosidase activities of *MG1655* and *VS111* in the absence of an inducer. Overnight cultures of *E. coli* cells were diluted 100-fold in LB and grown to an OD600 of 0.2, and assayed for β-galactosidase activities (Miller’s units). Black squares and circles represent β-galactosidase activities of *MG1655* and *VS111*, respectively. (**b**) Western blotting for the basal expression of β-galactosidase for *MG1655* (top panel) and *VS111* (bottom panel) in the absence of an inducer. Overnight cultures of *E. coli* cells were diluted 100-fold and grown to an OD600 of 0.5. The western blotting experiments were performed as described under Methods. (**c**) Plasmid pZXD133 was constructed as described in Methods. The *lacZ* and firefly luciferase (*luc*) genes were under the control of P_T7A1/O4_ and P_leu-500_ respectively. The winged triangles represent the Rho-indepepndent *E. coli rrnB T1* transcription terminators. (**d**) The β-galactosidase activities of *MG1655(DE3)*Δ*lacZ* and *VS111(DE3)*Δ*lacZ* carried plasmids pZXD133, pZXD145, or pZXD146 in the absence of an inducer. Plasmids pZXD133, 145, and 146 carrying one of the IPTG-inducible promoters (in the order of descending strength) P_T7A1/O4_, P_tac_, and P_lacUV5_, respectively, which control the expression of *lacZ.* Overnight cultures of *E. coli* cells were diluted 100-fold and grown to an OD600 of 0.2, and assayed for β-galactosidase activities. The black and open columns represent the β-galactosidase activities of MG1655 and VS111, respectively. The promoters that control the expression of *lacZ* are labeled. (e) Western blotting for the basal expression of β-galactosidase for MG1655(DE3)Δ*lacZ*/pZXD133 (top panel) and VS111(DE3)Δ*lacZ*/pZXD133 (bottom panel) in the absence of IPTG. The western blotting experiments were performed as described in Methods. (f) The basal expression of β-galactosidase in the absence of an inducer for *E. coli* strain *FL1130 (MG1655(DE3)*Δ*lacZ attnT7::P*_*T7A1/O4*_*-lacZ P*_*leu-500*_*-luc)* and *FL1131 (VS111(DE3)*Δ*lacZ attnT7::P*_*T7A1/O4*_*-lacZ P*_*leu-500*_*-luc)*. Overnight cultures of *E. coli* cells were diluted 100-fold and grown to an OD600 of 0.2, and assayed for β-galactosidase activities. Black squares and red circles represent β-galactosidase activities of *FL1130* and *FL1131*, respectively.

**Figure 4:**
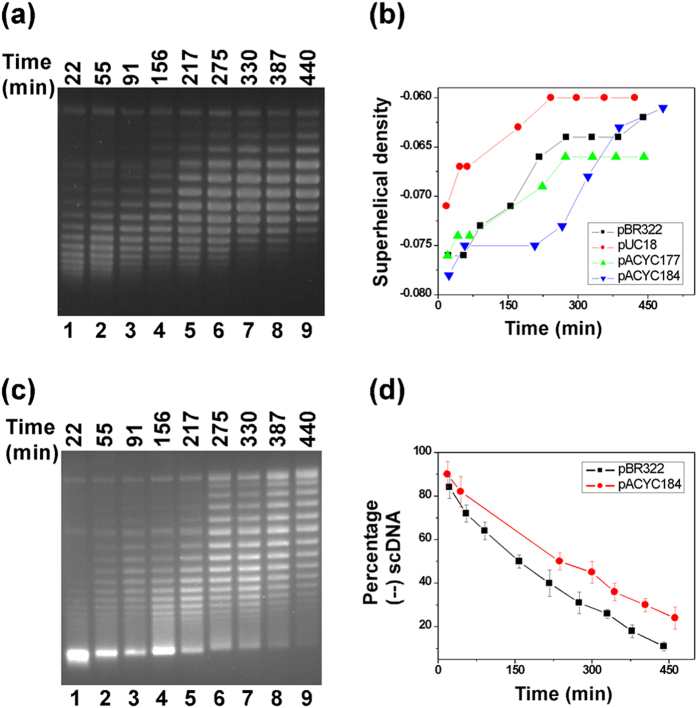
Time courses of DNA supercoiling status in MG1655 (**a**,**b**) and VS111 (**c**,**d**). Overnight cultures of *E*. *coli* cells carrying plasmids pBR322, pUC18, pACYC177, or pACYC184 were diluted 100-fold in LB and grown to the time points indicated. The DNA molecules were isolated using the alkaline lysis assays using the QIAprep Spin Miniprep Kit. The DNA samples were subjected to 1% agarose gel electrophoresis in the presence of 5 μg/mL of chloroquine. The DNA supercoiling densities were determined as detailed in Methods. The symbol of (–) represents the hypernegatively supercoiled DNA.

Although LacI is known to sterically repress transcription, it is not obvious that it should act as a topological barrier. One possibility is that LacI uses this unique functionality to trap free, unconstrained supercoils within the 401 bp DNA loop of the *lac* promoter, enhance the LacI DNA-binding affinity^18^,^19^,^20^,^26^, and therefore increase the promoter inhibition. In this way, LacI may modulate the expression of *lacZYA* in the *lac operon* across a wide range to respond to varying growth conditions. In other words, DNA supercoiling may regulate the basal expression of the *lac* operon *in vivo*. If this hypothesis is correct, the basal expression of *lacZ* (β-galactosidase) should be higher for a *wild-type E. coli* strain (*MG1655*) than for a *topA* mutant strain (*VS111*) since both chromosomal and plasmid DNAs are more (−) supercoiled in the mutant strain than in the *wild-type* strain^32^,^33^. Our results in Fig. 3a,b clearly demonstrated that the basal level expression of β-galactosidase in the absence of an inducer (such as IPTG) is indeed higher for *MG1655*. Intriguingly, the basal expression of β-galactosidase is higher for *E. coli* cells at the late exponential phase than that at the early exponential phase for both *MG1655* and *VS111* (Fig. 3a,b). If the above hypothesis is correct, DNA should be more (−) supercoiled at the early exponential phase than that at the late exponential phase. In order to answer this question, we examined the supercoiling status of several plasmids, i.e., pBR322, pUC18, pACYC177, and pACYC184 in *MG1655* and *VS111* at the early and late exponential phases. Our results are shown in Fig. 4. For *MG1655*, plasmid DNA templates are more (−) supercoiled at the early exponential phase than those at the late exponential phase. For instance, the superhelical density of plasmid pBR322 at the early exponential phase was determined to be −0.076 and diminished to −0.062 at the late exponential phase (Fig. 4a,b). For *VS111*, as expected, at the early exponential phase, 80–90% of plasmids pBR322 and pACYC184 became hypernegatively supercoiled (Fig. 4c,d), which is consistent with previously published results^32^,^34^. However, less than 50% of these two plasmids were hypernegatively supercoiled at the late exponential phase (Fig. 4c,d). These results suggest that a correlation exists between the DNA supercoiling status and the basal expression of β-galactosidase in *E. coli*.

In order to further study how DNA supercoiling regulates the basal expression of β-galactosidase, we constructed two *E. coli* strains *MG1655(DE3)*Δ*lacZ* and *VS111(DE3)*Δ*lacZ* in which *lacZ* was deleted from the chromosome using the λ Red recombination system^35^. Additionally, these two *E. coli* strains carry the lysogenic λ DE3 that harbors the *lacI* gene under the control of the strong lacI^q^ promoter to overexpress LacI. We also constructed three plasmids pZXD133, 145, and 146 carrying one of the IPTG-inducible promoters (in the order of descending strength) P_T7A1/O4_, P_tac_, and P_lacUV5_, respectively, which control the expression of *lacZ* (Fig. 3c). We transformed *MG1655(DE3)*Δ*lacZ* and *VS111(DE3)*Δ*lacZ* with these three plasmids and tested the β-galactosidase activities using Miller’s assay and Western blotting. Our results are shown in Fig. 3d,e. Similar to above results, the basal expression of β-galactosidase in the absence of an inducer is higher for *MG1655* than that for *VS111.* Since these IPTG-inducible promoters do not contain a CRP (catabolite repressor protein) binding site, these experiments ruled out the possibility that the enhanced basal expression of β-galactosidase is caused by the binding of the CRP-cAMP complex to the CRP binding site upstream from the *lac* promoter. Additionally, we inserted *lacZ* under the control of P_T7A1/O4_ in the *attTn7* site (84 min) of the chromosome of *MG1655(DE3)*Δ*lacZ* and *VS111(DE3)*Δ*lacZ* using a transposon Tn7-based method^36^ and tested the β-galactosidase activities using Miller’s assay. Again, our results (Fig. 3f) showed that the basal expression of β-galactosidase in the absence of an inducer is higher for *MG1655* than that for *VS111.*

## Discussion

**Figure 5:**
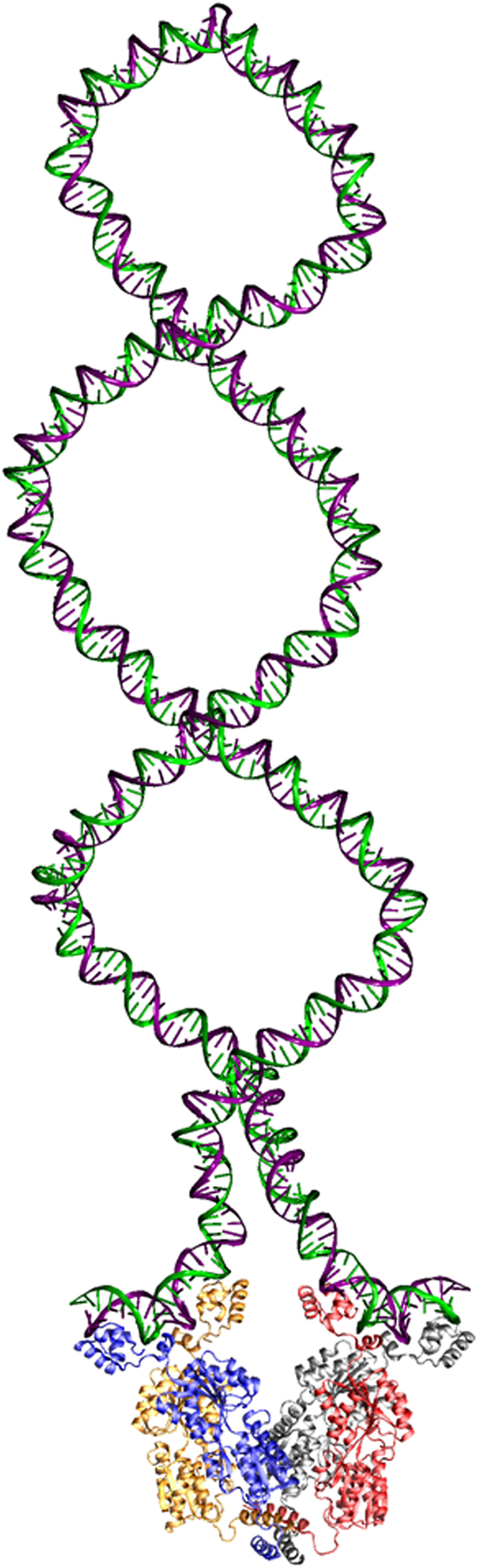
LacI tetramer simultaneously binds to *O1* & *O2* operators of the *lac* promoter and forms a topological barrier that constrains three (−) supercoils in the 401 bp DNA loop. This molecular model has been constructed as described under Methods and shows that it is feasible for the LacI-mediated topological barrier to constrain three (−) DNA supercoils to the 401 bp DNA loop.

In this article we demonstrate that *E. coli* LacI is able to form a topological barrier upon binding to the *O1*, *O2,* and *O3* operators of the *lac* promoter at their native positions and separate a supercoiled DNA molecule into two distinct topological domains: a large 4,300 bp domain and a small 401 bp domain. The small 401 bp domain is capable of constraining 3 (−) supercoils in the LacI-mediated DNA loop for the DNA template with a superhelical density of ~−0.06 (Fig. 2b and Table 2). These constrained supercoils increase the LacI’s DNA-binding affinity and therefore enhance its inhibition of the *lac* promoter in the absence of an inducer. We also demonstrate that the stability of LacI-mediated DNA topological barriers is proportional to its DNA binding affinity: the higher the DNA binding affinity, the more stable the LacI-mediated topological barrier (Supplementary Tables S1 and S2). Figure 5 shows a molecular model of the physical interactions between LacI and *lac* operators that play essential roles in the formation of the DNA topological barrier with 3 constrained (−) supercoils. As mentioned above, these constrained (−) supercoils are important for the *lac* operon. First, they bring *O1* and *O2* close to each other and enhance the probability of forming the LacI-mediated DNA loop. Second, these constrained (−) supercoils provide free energy to enhance the LacI’s DNA binding affinity and therefore form a highly stable repressorsome^37^ that efficiently inhibits the gene expression of the *lac* operon. DNA supercoiling appears to be an essential component of the classical *lac* operon in *E. coli*, a component that has been overlooked previously^1^,^4^,^12^. Nevertheless, it is important to understand what property or properties of LacI determine its capacity to form a topological barrier and block supercoiling diffusion. We showed previously^24^ that LacI is able to induce superhelicity (ΔLk) within the LacI-*lac O* complexes. This LacI-induced superhelicity may be essential for forming the DNA topological barrier and blocking supercoiling diffusion.

These experiments also show that DNA supercoiling plays an essential role in the regulation of the basal expression of the *lac* operon in *E. coli*. Not only did the *wild-type* strain *MG1655* express more β-galactosidase comparing with the isogenic *topA* strain *VS111* (Fig. 3a,b), but the expression level of β-galactosidase of *MG1655* and *VS111* is higher for cells at the early exponential phase than for those at the late exponential phase as well (Fig. 3a,b,e,f). The expression level is directly correlated with the DNA supercoiling status *in vivo* (Fig. 4). These results strongly support our hypothesis that DNA supercoiling modulates the basal expression of the *lac* operon, an effect allowing *E. coli* cells to sense the superhelical changes through the LacI-mediated topological barrier for different growth conditions.

*E. coli* is a single cell organism and has developed the ability to adapt rapidly to different environmental conditions, such as low nutrients, high osmotic pressure, and the change of temperature^38^. When the main carbon source glucose is abundant, *E. coli* cells generate sufficient ATP for normal functions and biosynthesis. In this case, DNA gyrase is fully active and drives the chromosomal DNA to more (−) supercoiled status^39^,^40^ (Fig. 4). The excess supercoils constrained in the 401 bp DNA loop by the LacI-mediated topological barrier promote the formation of a highly stable repressorsome that prevents the wasteful expression of *lacZYA* of the *lac* operon. However, when *E. coli* cells live in nutrient deficient environments, the ATP/ADP ratio or energy charge is low, which significantly reduces the supercoiling activities of DNA gyrase^41^,^42^. In this way, the DNA around the *lac* promoter is relaxed^39^,^40^ (Fig. 4), which weakens the binding of LacI to *lac* operators and therefore increases the basal level expression of *lacZYA.* This mechanism prepares *E. coli* cells to respond quickly to the presence of other carbon sources, such as lactose in the nutrient deficient environments. In this way, certain amounts of lactose is transported inside cells by lactose permease and converted to allolactose by β-galactosidase, the natural inducer of the *lac* operon. Our results in Fig. 3 are consistent with this explanation. These results also provide a reasonable explanation for the long-time observation in which DNA supercoiling enhances the DNA binding affinity of LacI to *lac* operators^18^,^19^ and promotes the formation of LacI-mediated DNA loop^20^,^21^,^22^. The *lac* operon of *E. coli* uses the LacI-mediated topological barrier to sense the environmental changes through sensing the DNA topological change around the *lac* operon. In this way, the cells can set the basal level of *lacZYA* expression.

In our recent publication, we demonstrated that bacterial phage λ utilizes supercoiling as a signal to decide whether the virus adopts a lytic or lysogenic life cycle when it infects an *E. coli* cell^31^. It is likely that the virus has evolved to respond to (–) DNA supercoils by facilitating the quiescent propagation through lysogenic life cycle during favorable growth conditions for bacteria^31^. In this article our results also suggest that *E. coli* genome may have evolved to regulate the basal expression of the *lac* operon by using LacI-mediated topological barrier as a sensor to detect the superhelical changes of chromosomal DNA during different growth conditions. It is possible that bacteria have adopted DNA topological barriers as a common mechanism to sense superhelical change within chromosomal DNA, the most dynamic epigenetic signal for transcription regulation. Since the nucleosomes are the basic packaging units for eukaryotes in which ~146 bp of DNA wrap in 1.67 left-handed superhelical turns around the histone octamer and (−) supercoils are constrains in the nucleosomes^43^, it is possible that also eukaryotes use topological barriers to regulate different biological functions, such as transcription and recombination.

## Methods

### Proteins, chemicals, and reagents

*E. coli* LacI and mutants were purified by the method of Chen and Matthews^44^ (*E. coli* strains containing the plasmid overexpressing LacI and mutants was kindly provided by K. S. Matthews at Rice University). Restriction enzymes Nt.BbvCI, Nb.BbvCI, Nb.BtsI, T4 DNA ligase, and *E. coli* DNA gyrase were purchased from New England Biolabs (Beverly, MA, USA). Isopropyl β-D-1-thiogalactopyranoside (IPTG) and o-nitrophenyl-β-D-galactoside (ONPG) were obtained from Sigma-Aldrich, Inc. (St. Louis, MO). All synthetic oligonucleotides were purchased from MWG-Biotech, Inc. (Huntsville, AL). γ-^32^P-ATP (3000 mCi/mmol) was obtained from PerkinElmer Life and Analytical Sciences (Shelton, CT).

### Plasmid DNA templates

All plasmids except pZXD133, pZXD145, and pZXD146 are derived from plasmid pACYC184. Construction of plasmid DNA templates sometimes required DNA fusions between non-complementary cohesive termini. In this scenario, cohesive ends were converted before ligation to blunt ends by incubation of the DNA fragments with T4 DNA polymerase in the presence of dNTPs. Plasmids pCB67, pCB73, pCB107, pCB109, pCB112, and pCB115 were described previously^25^. Plasmid pCB106 was constructed by the insertion of a 19 bp synthetic DNA fragment containing a nicking enzyme Nt.BbvCI recognition site into the unique EagI site of pCB73. Plasmid pCB108 was created by the insertion of the 378 bp BamHI-KpnI fragment of pCB106 carrying 8 tandem copies of *lac O1* operators to the unique AdhI site of pCB106. In this way, pCB108 contains 16 *lac O1* operators equally distributed between two locations. Plasmid pCB116 was constructed through the insertion of the 194 bp BamHI-KpnI fragment of pCB112 carrying 4 tandem copies of *lac O1* operators to the unique AdhI site of pCB112. In this way, pCB116 contains 8 *lac O1* operators equally distributed between two locations. Plasmid pCB110 was constructed by the insertion of the 19 bp synthetic DNA fragment described above into the unique EagI site of pCB67. Plasmid pCB126 (or pO1O1) was made by inserting a 46 bp synthetic oligonucleotide containing a *lac O1* operator into the unique AhdI site of pCB110. In this case, the shortest distance between the two-*lac O1* operators of pCB126 is 1.2 kb.

Plasmids pO1O2 and pO1O3 were constructed by inserting a 41 bp synthetic DNA oligonucleotide containing a *lac O2* or *O3* operator into the unique AhdI site of pCB110, respectively. Likewise, plasmid pO1Os was made by the insertion of a 40 bp synthetic DNA oligonucleotide containing a *lac Os* operator into the unique AhdI site of pCB110. Plasmid pO2O2 was created by the replacement of the 46 bp BamHI-BglII fragment of pO1O2 with a 31 bp synthetic DNA oligonucleotide carrying a *lac O2* operator. Plasmid pO3O3 was constructed by replacing the 46 bp BamHI-BglII fragment of pO1O3 with a 31 bp synthetic DNA oligonucleotide carrying a *lac O3* operator. Plasmid pOsOs was created by replacing the 46 bp BamHI-BglII fragment of pO1Os with a 31 bp synthetic DNA oligonucleotide carrying a *lac Os* operator. Plasmid pO3O1O2 was constructed in two steps. Step 1 is to insert a 568 bp PCR product containing *lac O1*, *O2*, and *O3* operators at their native positions on the chromosome into the BamHI-KpnI sites of pYZX43F to generate plasmid pO1O2O3-1. In the second step, a nicking enzyme Nt.BbvCI recognition site between the *lac O1* and *O2* operators was created to yield plasmid pO3O1O2. Plasmids pO1O2n and pO3O2n were made by PCR-directed site-mutagenesis to eliminate the *lac O3* or *O2* operators of pO3O1O2, respectively. Plasmids pOsOs401 and pOsOs493 were, respectively, created through converting the *lac* operators of pO1O2n and pO3O2n into *lac Os* operators by PCR-based site-directed mutagenesis.

Plasmids pZXD133, pZXD145, and pZXD146 are derivative of pBR322 and were constructed in several steps. Plasmid pZXD64 was constructed by introducing a unique AgeI site into the upstream region of the *tet* gene of pZXD14^45^ using PCR-based, site-directed mutagenesis. Then, the *tet* gene between the unique AgeI and BsmI sites of pZXD64 was replaced by a 3,068 bp *lacZ* gene DNA fragment of plasmid pYC2/CT/lacZ (Life Technologies, Grand Island, NY) to generate pZXD65. Next, four Rho-independent *E. coli rrnB* T1 terminators from plasmid pLUC1 were inserted into XbaI site of pGL3 (Promega Corporation, Wisconsin, WI) to yield pZXD67. A 2,511 bp HindIII-SpeI DNA fragment of pZXD67 carrying a modified firefly (*Photinus pyralis*) *luciferase* gene (the codon usage was optimized for mammalian cells) and four Rho-independent *E. coli rrnB* T1 terminators was inserted between the HindIII and SpeI sites of pZXD65 to produce pZXD70. Plasmid pZXD74 was created by silently removing the EcoRI site in the downstream region of *lacZ* gene of plasmid pZXD70 without changing the open reading frame of *lacZ* gene using PCR-based, site-directed mutagenesis. Plasmid pZXD76 was generated after a XbaI site was inserted into the downstream region of the *luciferase* gene of plasmid pZXD74. Then, a 1,688 bp DNA fragment of pZE15luc carrying a firefly (*Photinus pyralis*) *luciferase* gene (the codon usage was optimized for bacterial cells) was inserted into the HindIII and XbaI sites of pZXD76 to yield pZXD77. Plasmid pZXD97 was created by inserting a 72 bp synthetic deoxyoligonucleotide containing *leu-500* promoter and a unique BamHI site into the HindIII and EcoRI sites of pZXD77. pZXD99 was produced by inserting a 53 bp synthetic deoxyoligonucleotide into the BamHI and EcoRI sites of pZXD97. Plasmid pZXD105 was constructed by inserting a 55 bp synthetic DNA oligonucleotide containing the strong IPTG-inducible *E. coli* T7A1/O4 promoter into the EcoRI and XhoI sites of pZXD99. In this way, the T7 promoter was replaced by the T7A1/O4 promoter. Plasmid pZXD133 was made by the insertion of a 3,093 bp PCR product containing the *lacZ* gene amplified from *MG1655* genomic DNA into the AgeI and BsmI sites of pZXD105. In this case, the codon usage of *lacZ* was optimized for *E. coli* cells. Plasmid pZXD106 was constructed by inserting an 85 bp synthetic DNA oligonucleotide containing the tac promoter into the EcoRI and XhoI sites of pZXD99. In this way, the T7 promoter was replaced by P_tac_. Plasmid pZXD145 was created by the insertion of a 3,093 bp PCR product containing the *lacZ* gene amplified from *MG1655* genomic DNA into the AgeI and BsmI sites of pZXD106. Plasmid pZXD107 was made by inserting an 87 bp synthetic DNA oligonucleotide containing the lacUV5 promoter into the EcoRI and XhoI sites of pZXD99. In this way, the T7 promoter was replaced by P_lacUV5_. Plasmid pZXD146 was constructed by the insertion of a 3,093 bp PCR product containing the *lacZ* gene amplified from *MG1655* genomic DNA into the AgeI and BsmI sites of pZXD107.

### Bacterial strains

*Escherichia coli* strains *MG1655* [F^−^, λ^−^, *rph-I*] and *VS111* [F^−^, λ^−^*, rph-I,* Δ*topA*] were obtained from the *Coli* Genetic Stock Collection/*E. coli* Genetic Resource Center (CGSC) at Yale University. *MG1655(DE3)* and *VS111(DE3)* were described previously^34^. *E. coli* strains *FL1130* (*MG1655(DE3)* Δ*lacZ attnT7::P*_*T7A1/O4*_*-lacZ P*_*leu-500*_*-luc*) and *FL1131* (*VS111(DE3)* Δ*lacZ attnT7::P*_*T7A1/O4*_*-lacZ P*_*leu-500*_*-luc*) were constructed in two steps. First, *MG1655(DE3)* Δ*lacZ* and *VS111(DE3)* Δ*lacZ* were constructed using the λ Red recombination system^35^. In the next step, using a Tn7-based site-specific recombination system^36^, a 5.1 kb DNA fragment carrying the divergently coupled P_leu-500_ and P_T7A1/O4_ promoters with the *luc* and *lacZ* genes was inserted to the *attTn7* site of the *E. coli* chromosome^46^ (84 min of the chromosome) to generate *FL1130* and *FL1131*. In both strains, the IPTG-inducible P_T7A1/O4_ controls the expression of β-galactosidase.

### The DNA-nicking method

The DNA-nicking method was described previously^25^ with some modifications. Briefly, a typical DNA-nicking reaction mixture (320 μL) contained 20 mM Tris-acetate (pH 7.9 at 25 °C), 10 mM magnesium acetate, 1 mM DTT, a negatively supercoiled DNA template, and LacI. Where specified, 1 mM of IPTG was also added to the DNA-nicking assays. All components were assembled on ice and incubated for 30 min at 37 °C. After the incubation, the supercoiled DNA templates were digested by either Nt.BbvCI or Nb.BbvCI at 37 °C for 30 min or various times. Then, a large excess of a double-stranded oligonucleotide containing Nt.BbvCI recognition site were added to the reaction mixtures to inhibit the restriction enzyme activities. The nicked DNA templates were ligated by T4 DNA ligase in the presence of 1 mM of ATP at 37 °C for 5 min and the reactions were terminated by extraction with an equal volume of phenol. The DNA samples were precipitated with ethanol and dissolved in 25 μL of 10 mM Tris-HCl buffer (pH 8.5). The linking number of the ligated DNA products was determined with 1% agarose gel electrophoresis in the absence or presence of 0.5 μg/ml of chloroquine and calculated from the gel images stained with SYBR Gold using KODAK 1D Image Analysis Software.

### Determining supercoiling density of plasmid DNA templates

3 μg of supercoiled plasmid pBR322, pUC18, pACYC177, or pACYC184 was relaxed by human DNA topoisomerase I in the presence of various concentrations of ethidium bromide at 37 °C in 20 mM Tris-acetate (pH 7.9), 50 mM KAc, 10 mM Mg(Ac)_2_, and 1 mM DTT for one hour. Subsequently, the relaxation reaction was stopped by addition of an equal volume of phenol. The topological status of each DNA preparation was analyzed by electrophoresis in a 1% agarose gel in 1 × TAE buffer (40 mM Tris-acetate (pH 7.8) and 1 mM EDTA) containing different concentrations of chloroquine. After electrophoresis, agarose gels were stained with ethidium bromide, destained, and photographed under UV light. The DNA linking number change (ΔLk) was determined by analyzing the distributions of the topoisomers in these gel images and the supercoiling density (σ) was calculated using the following equation:


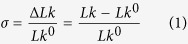


where Lk^0^ and Lk represent the DNA linking number for the relaxed and the supercoiled DNA, respectively.

### Electrophoretic Mobility Shift Assay (EMSA)

EMSA experiments were used to determine the apparent DNA binding constant of LacI and mutants. DNA oligomers containing a DNA-binding site of one of the DNA-binding proteins (the EcoRV fragment of the pBend2 derivatives) were labeled with ^32^P at 5′ termini by T4 polynucleotide kinase in the presence of γ-^32^P-ATP. The protein-DNA complexes were formed by addition of appropriate amounts of the protein to a solution containing 0.1 nM of ^32^P-labeled DNA in the 1 × DNA-binding buffer containing 20 mM Tris-HCl (pH 8.0), 200 mM NaCl, 0.5 mM EDTA, 1 mM DTT, 5 mM MgCl_2_, and 5% glycerol. After equilibration for 60 min at 22 °C, the samples were loaded on a 8% native polyacryamide gel in 0.5 × TBE buffer (0.045 M Tris-Borate (pH 8.3) and 1 mM EDTA) to separate free and bound DNA. The gels were subsequently dried and visualized by autoradiography or quantified using a Fuji FLA 3000 image analyzer. The radioactivity of the free and bound DNA was determined and used to calculate the binding ratio (R), which is equal to the ratio of the radioactivity of the bound DNA divided by the sum of the radioactivity of the bound and free DNA. The apparent DNA binding constant (K_app_) was obtained by nonlinear-least-squares fitting the following equation using the program Scientist.


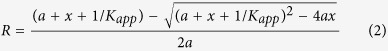


where a and x represent the total DNA and the total protein concentration, respectively.

### Atomic Force Microscopy

The LacI-DNA samples were prepared according to the DNA-nicking method as described above. After the supercoiled DNA templates were digested by Nt.BbvCI at 37 °C for 30 min, the LacI-DNA complexes (10 μL) were deposited on a poly-L-ornithine-coated mica surface and incubated for 2 min at room temp. The droplet was rinsed away with 0.4 mL HPLC water and dried gently with compressed air. Images were acquired with a NanoScope MultiMode AFM microscope (Digital Instrument, Santa Barbara, CA) operated in tapping mode using a 50–60 mV oscillation amplitude of uncoated, etched silicon tips with a resonance frequency of 75 kHz (NSC18, MirkoMasch, San Jose, CA^47^). Areas of 1 × 1 μm^2^ were scanned at a rate of 1.2 Hz and a resolution of 512 × 512 pixels. The DNA contour lengths were estimated by using Image Analysis Software ImageJ. The LacI-mediated 401 bp DNA loops were identified by measuring the DNA contour lengths of these relaxed DNA loops and by comparing the size and volume of the LacI molecules that form the DNA loops with our previously published results^25^.

### The expression of β-galactisidase

The expression level of β-galactosidase was measured by Miller’s assay as described^48^. Briefly, 100 mL of LB was inoculated with 1 mL of overnight bacterial cell culture until OD600 reaches ~0.2. 100 μL of bacterial cell culture was mixed with 900 μL of Z-buffer (60 mM Na_2_HPO_4_, 40 mM NaH_2_PO_4_, 10 mM KCl, 1 mM MgSO_4_, and 50 mM β-mercaptoethanol). Cells were lysed with 60 μL of chloroform and 30 μL of 0.1% SDS. After cell lysates were equilibrated at 30 °C for five minutes, 200 μL of 4 mg/mL ONPG was added to the cell lysates. After additional 15 min incubation at 30 °C, reactions were stopped by addition of 500 μL of 1 M Na_2_CO_3_. After cell debris was removed by centrifugation at 13,000 rpm for 1 min, OD420 and OD550 were measured in a Cary 50 spectrophotometer. β-Galactosidase activities (E) were calculated using the following equation:


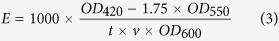


where *t* and *v* represent reaction time and cell culture volume, respectively.

### Western blotting experiments

Western blotting experiments were used to monitor the basal level expression of β-galactosidase in different *E. coli* strains. Total protein purified from *E. coli* cells was analyzed by electrophoresis in a 10% SDS-PAGE gel and electrophoretically transferred to a 0.45 nm nitrocellulose membrane. The membrane blot was then blocked with a solution containing 5% nonfat skim milk in TBST (50 mM Tris-HCl, pH 8.0, 138 mM NaCl, 2.7 mM KCl, and 0.05% Tween-20) for 45 min at room temperature and incubated with the primary antibody, β-galactosidase antibody (Thermofisher Scientific, Inc.), diluted 1:1000 in TBST solution overnight at 4 °C. After the overnight incubation, the membrane blot was washed three times with TBST and blocked with a solution containing 5% nonfat skim milk in TBST for 15 min at room temperature. Membranes were incubated with secondary antibodies in blocking buffer at 1:20,000 for fluorescently conjugated antibodies purchased from Li-Cor Biosciences. Membranes were again washed three times for five minutes in 1 × TBST. Western blots were developed using fluorescence detection using the LI-COR Odyssey CLx near infrared scanner.

### Molecular modeling

The DNA-loop was created with the GraphiteLifeExplorer modeling tool^49^. VMD software^50^ was used to patch and render the DNA-loop structure with the LacI-DNA complex (PDB id 1Z04), which was originally modeled by patching several NMR-structures of fragments of LacI and DNA^51^. Three (−) DNA supercoils were introduced to the DNA loop to match our experimental results for the LacI-mediated 401 bp DNA loop.

## Additional Information

**How to cite this article**: Fulcrand, G. *et al.* DNA supercoiling, a critical signal regulating the basal expression of the *lac* operon in *Escherichia coli*. *Sci. Rep.* **6**, 19243; doi: 10.1038/srep19243 (2016).

## Supplementary Material

Supplementary Information

## Figures and Tables

**Table 1:** Half-lives (t_1/2_) of LacI-mediated, DNA topological barriers for plasmids containing *lac O1* operators in two different locations.

ploperators in two different locations.asmid^a^	Number of lacO1 in each location	t_1/2_(min)	
		–IPTG^b^	+IPTG^b^
pCB126	1	0.87 ± 0.33	—
pCB115	2	112 ± 28	0.38 ± 0.18
pCB116	4	120 ± 39	53 ± 14
pCB108	8	168 ± 51	225 ± 79
pCB109	16	134 ± 57	242 ± 82

^a^All plasmids contain tandem copies of *lac O1* operator in two different locations.

^b^The half-life (t_1/2_) was determined according to the procedure as described under Methods.

**Table 2:** Sizes of the DNA loops and the number of supercoils constrained by the LacI-mediated DNA topological barrier.

Plasmid	Size of the DNA-loop^a^	t_1/2_ (min)	Supercoils constrained in the DNA-loop
pOsOs493	493 bp	56.1 ± 7.6	3.79 ± 0.31
pOsOs401	401 bp	46.8 ± 7.9	2.5 ± 0.27
pO3O2O1	493 bp	1.10 ± 0.15	3.38 ± 1.17
pO3O2n	493 bp	0.35 ± 0.02^b^	3.45 ± 1.10
pO1O2n	401 bp	1.41 ± 0.24	2.77 ± 0.65

^a^The size of the DNA-loop refers to the shorter DNA-loop between the two *lac* operators.

^b^The t_1/2_ using pO3O2n was estimated according to the DNA nicking method as described under Methods. Considering the efficiency of restriction digestion and DNA ligation reactions, the standard deviation may be much bigger than that reported in this table.

**Table 3:** DNA contour lengths of pOsOs401 in the presence or absence of LacI.

DNA domain	Measured DNA contour length		DNA sequence length
	nm	bp	bp
Full length	1584.4 ± 78.2	4660 ± 230^a^	4595
Rx domain^b^	1441.2 ± 50.3	4239 ± 148^a^	4194
Sc domain^c^	140.9 ± 23.8	414 ± 70^a^	401

^a^The measured DNA contour lengths in bp were calculated with the assumption of the standard B-form DNA for the plasmid; i.e., using a rise of 0.34 nm per base pair.

^b^Rx domain represents the relaxed DNA domain.

^c^Sc domain represents the supercoiled domain.
