# Supplementary material for: DNA supercoiling, a critical signal regulating the basal expression of the lac operon in Escherichia coli
Source: Sci Rep. 2016 Jan 14;6:19243. doi: 10.1038/srep19243 (PMC4725879; doi:10.1038/srep19243)
Supplement: Supplementary Information [file srep19243-s1.pdf]

Supplementary Information

DNA supercoiling, a critical signal regulating the basal expression of the *lac* operon in *Escherichia coli*

Geraldine Fulcrand[1,2], Samantha Dages[1,2], Xiaoduo Zhi[1,2], Prem Chapagain[1,3], Bernard S. Gerstman[1,3], David Dunlap[4], and Fenfei Leng[1,2,*]

[1]Biomolecular Sciences Institute, [2]Department of Chemistry & Biochemistry, [3]Department of Physics, Florida International University, Miami, FL 33199; and [4]Department of Physics and Cell Biology, Emory University, Atlanta, GA 30322

* To whom correspondence should be addressed: Department of Chemistry & Biochemistry, Florida International University, 11200 SW 8th Street, FL 33199. Tel: 305-348-3277; Fax: 305-348-3772; E-mail: lengf@fiu.edu

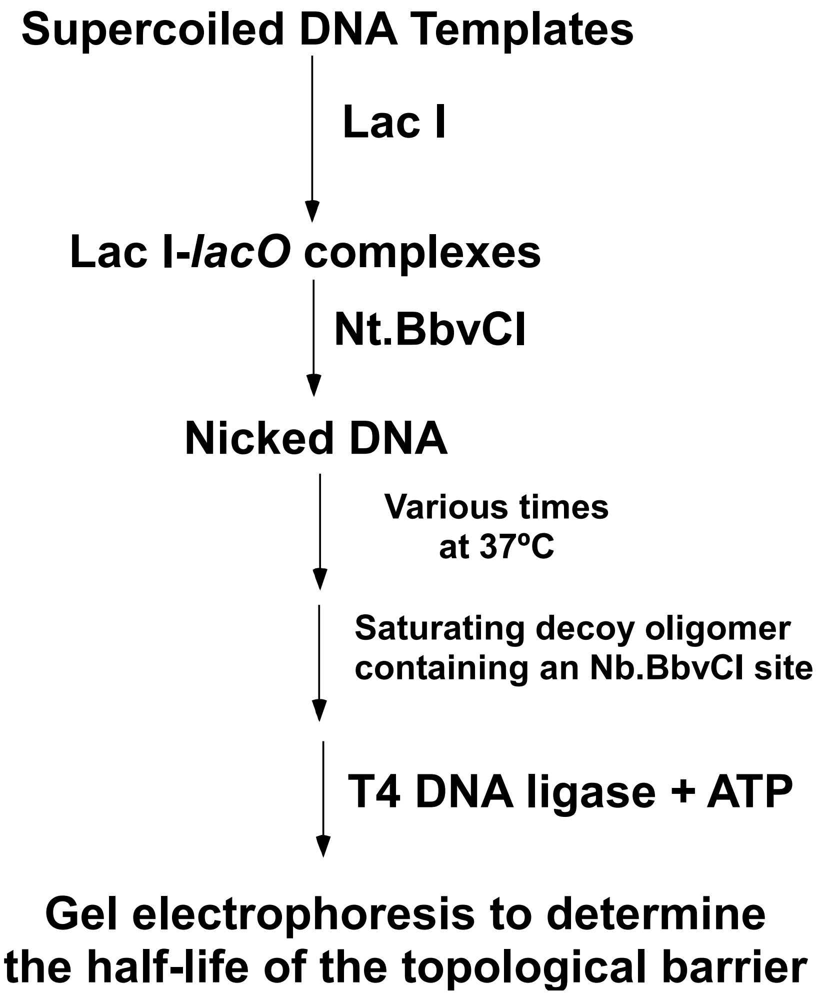

Supplementary Fig. S1. The DNA nicking method to determine the stability of DNA topological barriers stemming from LacI-*lacO* DNA-looping complexes. A (-) supercoiled DNA template, which contains recognition sites for the nicking endonucleases Nt.BbvCI & Nb.BtSI and *lac O1* operators in two different locations, was used in these experiments. After *E. coli* LacI binds to the operators to form LacI-*lacO1* DNA-looping complexes, the DNA template was digested by Nt.BbvCI. A large excess of oligonucleotides containing an Nt.BbvCI recognition site was then added to the reaction mixture to inhibit the enzyme activities. After ligation by T4 DNA ligase and phenol extraction, the linking number change (ΔLk) of the DNA molecule was determined by gel electrophoresis.

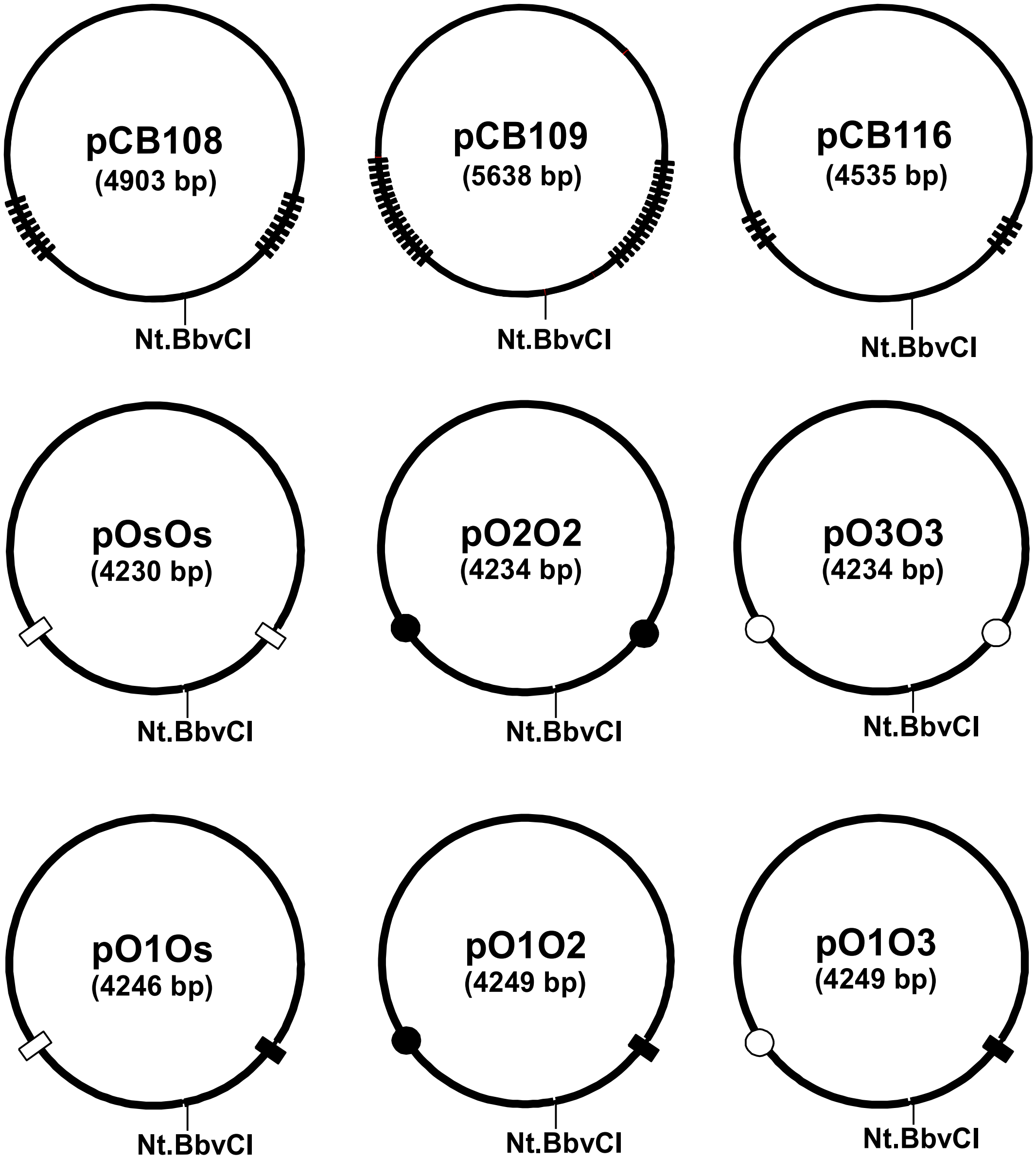

Supplementary Fig. S2. Plasmids containing different *lac* operators. The restriction enzyme sites for Nt.BbvCI are shown. The closed rectangle represents a *lac O1* operator. The restriction enzyme site for Nt.BbvCI is shown. The closed rectangles, the open rectangles, closed circles, and open circles represent *lac O1*, *Os*, *O2* and *O3* operators, respectively.

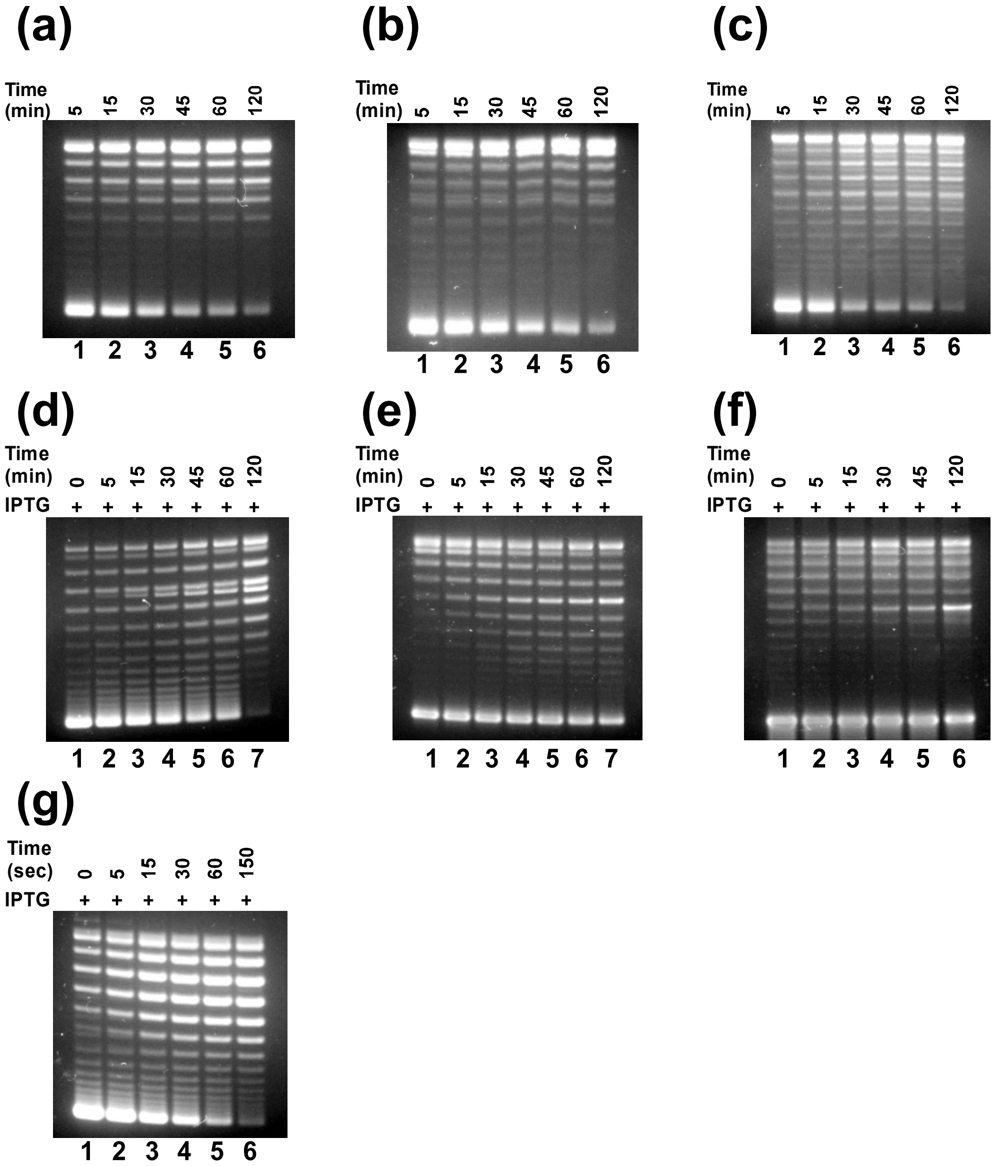

Supplementary Fig. S3. Time courses of DNA supercoiling diffusion in the presence of LacI for plasmids pCB116 (a), pCB108 (b), and pCB109 (c) in the absence of IPTG and for plasmids pCB116 (d), pCB108 (e), pCB109 (f), and pCB115 (g) in the presence of IPTG. The DNA-nicking assays were performed as described under Materials and Methods. Each reaction mixture (320 μL) contained 0.156 nM of plasmid DNA templates, 2.5 nM of LacI, and 12 units of Nt.BbvCI. The reaction mixtures were incubated at 37 °C for the time indicated. Then, a large excess of a double-stranded oligonucleotide containing an Nt.BbvCI recognition site were added to the reaction mixtures to inhibit the restriction enzyme activities. The nicked DNA templates were ligated by T4 DNA ligase in the presence of 1 mM of ATP at 37 °C for 30 min and the reactions were terminated by extraction with an equal volume of phenol. The plasmid DNA molecules were isolated and subjected to agarose gel electrophoresis in the absence of chloroquine. For plasmid pCB115, 16 units of Nt.BbvCI were used.

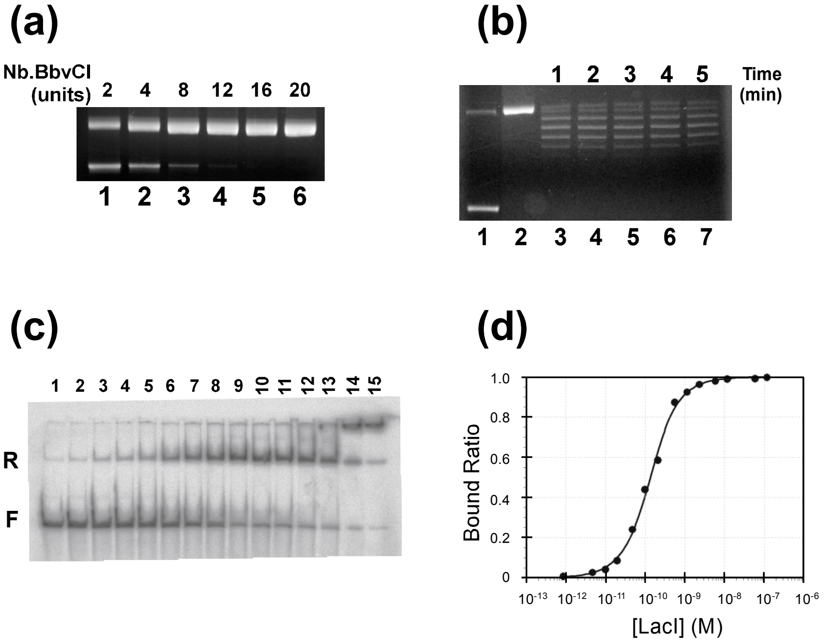

Supplementary Fig. S4. (a) The nicking endonuclease Nt.BbvCI was able to rapidly digest plasmid pCB126. The digestion of pCB126 using different amounts of Nt.BbvCI in 320 μL of 1×NEB buffer 4. The restriction enzyme digestion assays were performed as described under Materials and Methods. Lanes 1 to 6 contained DNA samples isolated from the reaction mixtures digested by 2, 4, 8, 12, 16, 20 units of Nt.BbvCI for 30 seconds, respectively. **(b) The nicked DNA templates were efficiently ligated by T4 DNA ligase in the DNA nicking assays.** The DNA nicking assays in the absence of LacI were performed as described under Materials and Methods. Each reaction mixture (320 μL) contained 0.156 nM of plasmid pCB126 and 16 units of Nt.BbvCI. The reaction mixtures were incubated at 37 °C for 5 minutes. Then, a large excess of a double-stranded oligonucleotide containing an Nt.BbvCI recognition site were added to the reaction mixtures to inhibit the restriction enzyme activities. The nicked DNA templates were ligated by T4 DNA ligase in the presence of 1 mM of ATP at 37 °C for the time indicated and the reactions were terminated by extraction with an equal volume of phenol. The plasmid DNA molecules were isolated and subjected to agarose gel electrophoresis in the absence of chloroquine. **(c) Gel mobility shift analysis: titration of *lac* O_1 DNA with wild type LacI.** Addition of LacI to a solution containing the free ^{32}P-labeled *lac O1* fragment (*F*) resulted in the formation of a 1:1 repressor-operator complex (*R*). All reactions were carried out in 10 mM Tris-HCl, pH 8.0, 1 mM EDTA, 250 mM KCl, and 0.1 mg/ml BSA. All samples contained 5.0×10^{-11} M *lac* O1 DNA. Lanes 2 to 15 also contained 1.2×10^{-13}, 1.2×10^{-12}, 2.4×10^{-12}, 6.0×10^{-12}, 1.2×10^{-11}, 2.4×10^{-11}, 6.0×10^{-11}, 1.2×10^{-10}, 2.4×10^{-10}, 6.0×10^{-10}, 1.2×10^{-9}, 2.4×10^{-9}, 6.0×10^{-9}, 1.2×10^{-8}, 6.0×10^{-8}, and 1.2×10^{-7} M LacI (tetramer), respectively. High concentrations of LacI cause formation of other bands of LacI-operator complexes (lanes 14 and 15) as reported previously (*3*). **(d) *A* representative isotherm for the binding of *LacI* to the *lac O1* DNA fragment.** The fraction of *lac O1* DNA bound (Y) is plotted as a function of the $\log_{10}$ of the free [LacI]. The solid curves were calculated using values of $K_{obs} = 1.2\times10^{10}$ M^{-1} obtained by fitting the binding data.

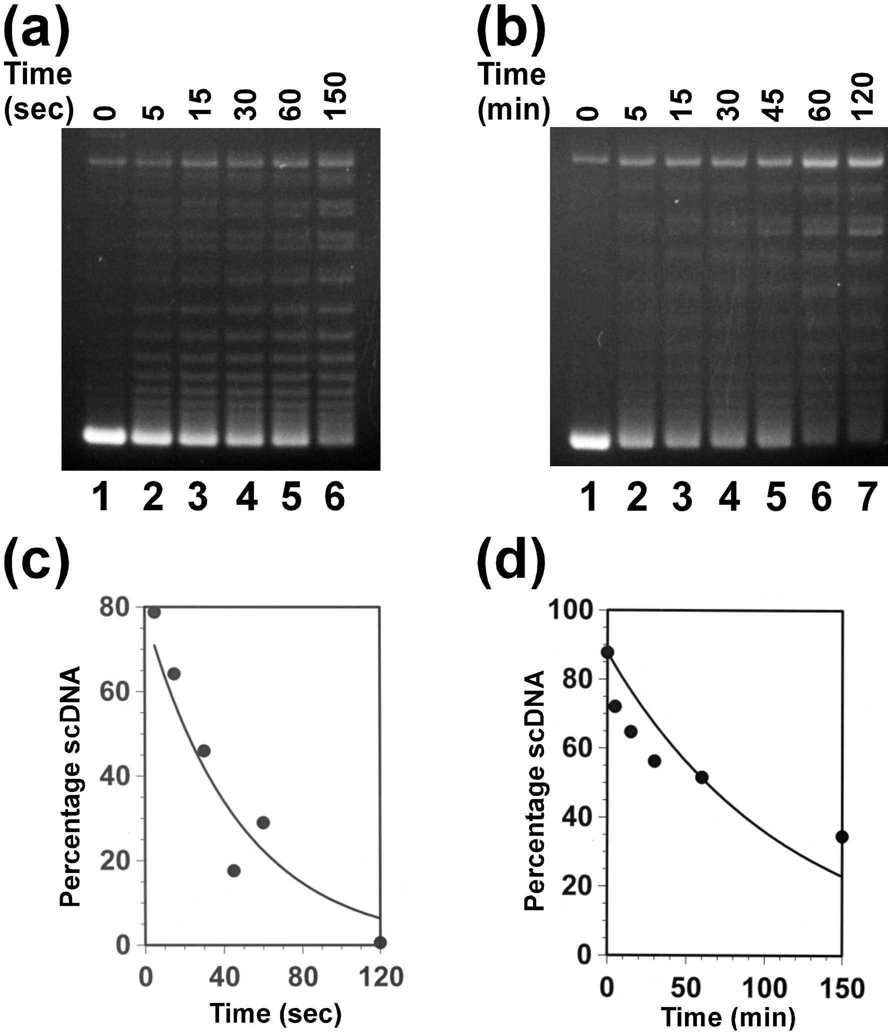

Supplementary Fig. S5. Time courses of DNA supercoiling diffusion in the presence of LacI for plasmids pO1O1 (a, c) and pOsOs (b, d). The DNA-nicking assays were performed as described under Materials and Methods. Each reaction mixture (320 μL) contained 0.156 nM of pO1O1 or pOsOs, 2.5 nM of LacI, and 16 units of Nt.BbvCI. The reactions were incubated at 37 °C for the time indicated. Then a large excess of a double-stranded oligonucleotide containing an Nt.BbvCI recognition site were added to the reaction mixture to inhibit the restriction enzyme activities. The nicked DNA templates were ligated by T4 DNA ligase in the presence of 1 mM of ATP at 37 °C for 5 min and the reactions were terminated by phenol extraction. The DNA molecules were isolated and subjected to agarose gel electrophoresis **(a, b)**. **(c, d) Quantification analysis of the time course.** The percentage of supercoiled DNA was plotted against the reaction time. The curve was generated by fitting the data to a 1st-order rate equation to determine the rate constant and $t_{1/2}$.

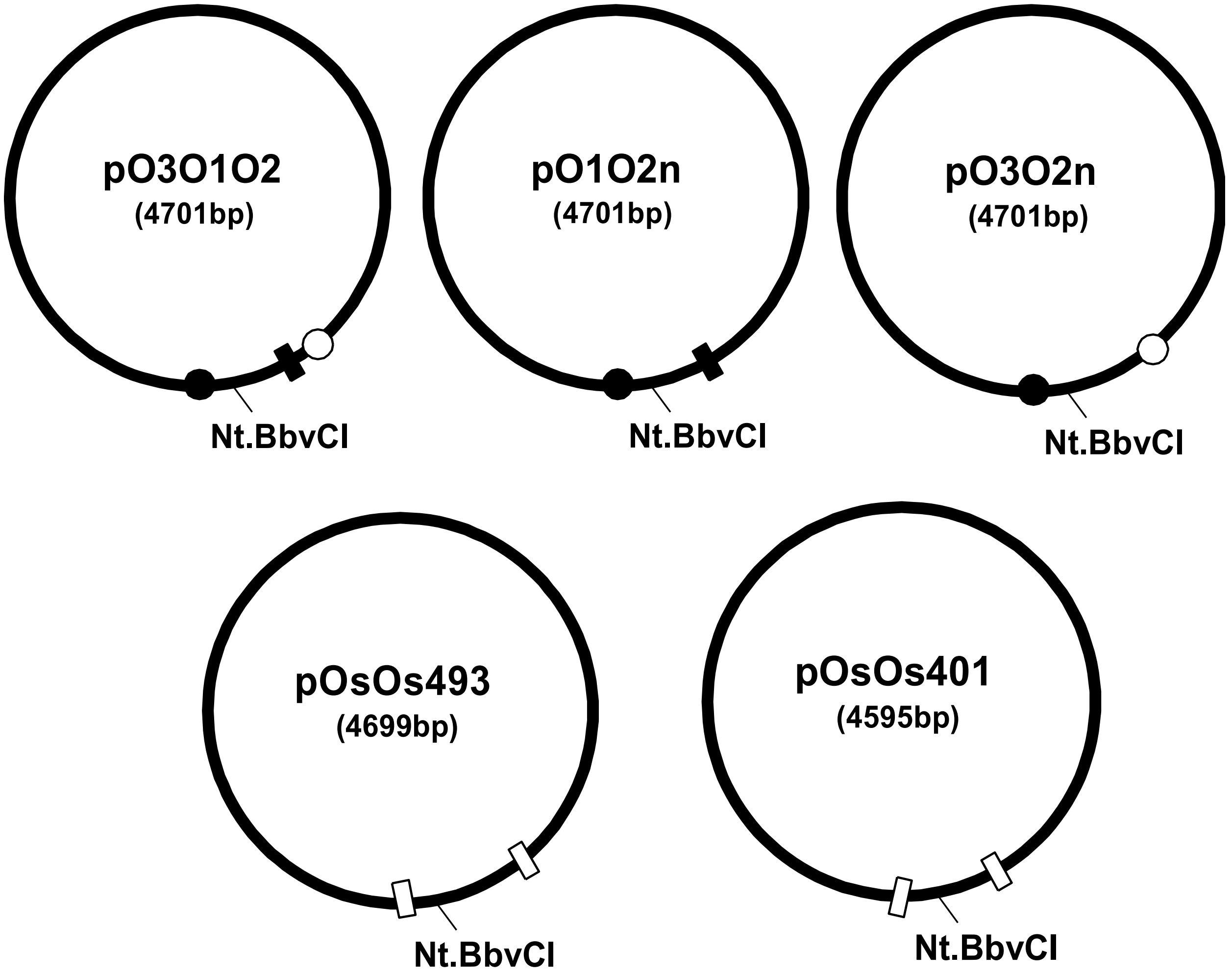

Supplementary Fig. S6. Plasmids containing *lac* operators at their native position of the *lac* promoter. The restriction enzyme site for Nt.BbvCI is shown. The rectangles, open rectangles, circles, and open circles represent *lac O1*, *Os*, *O2* and *O3* operators, respectively.

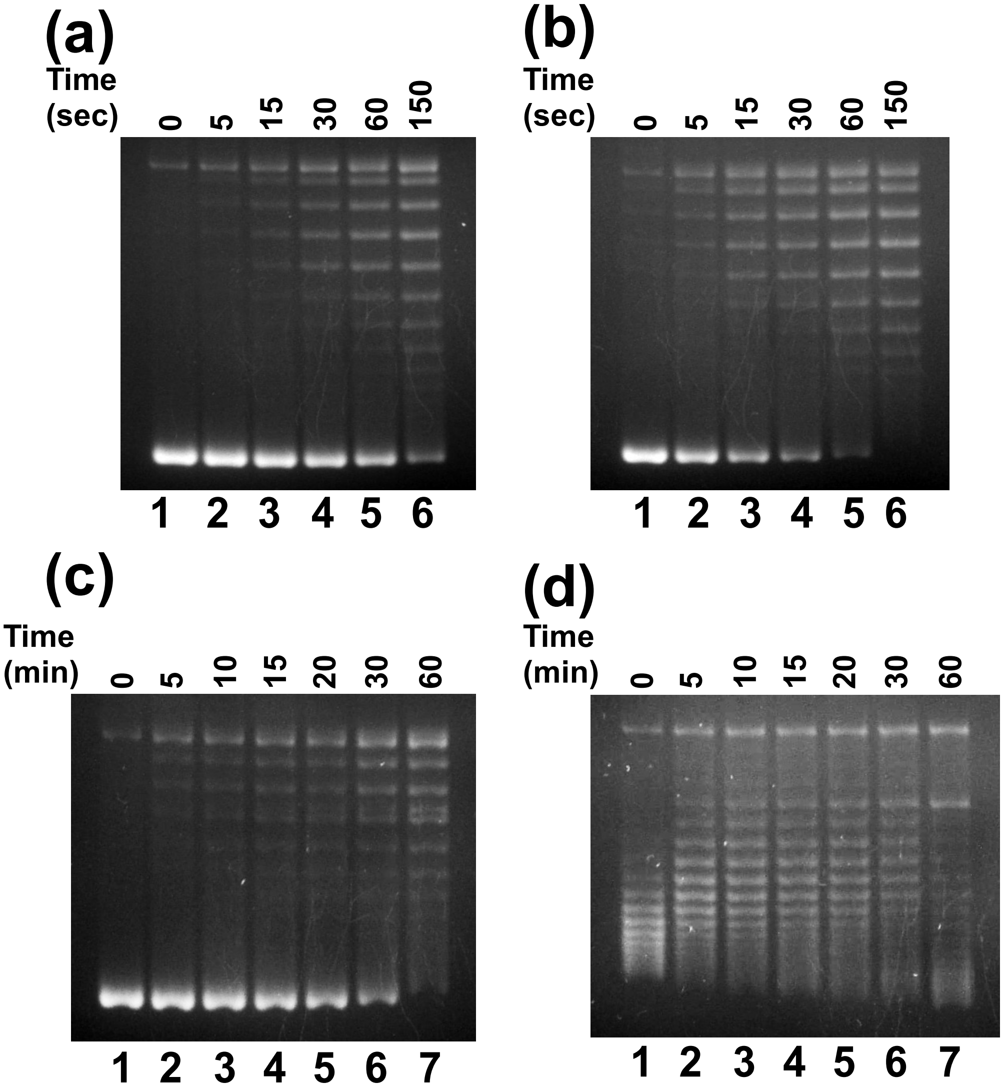

Supplementary Fig. S7. LacI was able to form a DNA topological barrier upon binding to the *lac* operators in their native position and divide supercoiled plasmid DNA molecules into two independent topological domains. (A) The DNA-nicking assays (time course) using supercoiled plasmid pO1O2n (σ = ~-0.06) were performed as described in Fig. 2B. The reaction mixtures in the presence of LacI were incubated at 37ºC for the time indicated. The DNA topoisomers were isolated and subjected to agarose gel electrophoresis in the absence of chloroquine. **(B)** The DNA-nicking assays (time course) using supercoiled plasmid pO2O3n (σ = ~-0.06) were performed as described in Supplementary Fig. 1B. **(C, D)**. The DNA-nicking assays (time course) using supercoiled plasmid pOsOs401 (σ = ~-0.06) were performed as described in Supplementary Fig. S1. The reaction mixtures in the presence of LacI were incubated at 37ºC for the time indicated. The DNA topoisomers were isolated and subjected to agarose gel electrophoresis in the absence **(C)** or presence **(D)** of 1.5 μg/mL chloroquine.

Supplementary Table S1. Half-lives ($t_{1/2}$) of LacI-mediated, DNA topological barriers for plasmids containing one lac operator (*Os, O1, O2, or O3*) in two different locations.

Plasmid	LacI and mutants	*lac* operators	$t_{1/2}$
pOsOs	WT	*Os, Os*	29.1±4.6 min
pO1Os	WT	*O1, Os*	68.3±16.3 sec
pO1O1	WT	*O1, O1*	51.6±9.6 sec
pO1O2	WT	*O1, O2*	24.4±5.8 sec[a]
pO2O2	WT	*O2, O2*	21.2±2.1 sec[a]
pO1O3	WT	*O1, O3*	5.8±2.2 sec[a]
pO3O3	WT	*O3, O3*	5.5±1.2 sec[a]
pOsOs	58+1	*Os, Os*	17.1±2.6 min
pOsOs	60+1	*Os, Os*	9.4±0.7 min
pOsOs	60+2	Os, Os	5.8±0.8 min
pOsOs	60+3	*Os, Os*	4.9±0.9 min

[a]The $t_{1/2}$ using pO3O2n was estimated according to the DNA nicking method as described under Materials and Methods. Considering the efficiency of restriction digestion and DNA ligation reactions, the standard deviation may be much bigger than that reported in this table.

Supplementary Table S2. Apparent DNA binding constants (K_{obs}, M^{-1}) for LacI and mutants binding to *lac O1, O2, O3,* and *Os* DNA fragments determined by gel mobility shift assay

	lac O1	*lac Os*	*lac O2*	*lac O3*
wild-type	1.2×10^{10}	5.7×10^{10}	2.3×10^{9}	3.1×10^{7}
Gly58+1	5.3×10^{9}	9.2×10^{9}	2.9×10^{8}	ND[a]
Gly60+1	2.4×10^{9}	5.2×10^{9}	5.8×10^{8}	ND[a]
Gly60+2	1.0×10^{9}	5.8×10^{9}	1.4×10^{8}	ND[a]
Gly60+3	1.0×10^{9}	2.4×10^{9}	6.2×10^{8}	ND[a]

The DNA binding constants were determined according to the procedure as described under Materials and Methods. [a]ND represents not determined.
